# Effects and Outcomes of Interferon Treatment in Japanese Hepatitis C Patients

**DOI:** 10.1186/1471-230X-12-139

**Published:** 2012-10-12

**Authors:** Kazumi Yamasaki, Mayumi Tomohiro, Yumiko Nagao, Michio Sata, Toshiaki Shimoda, Kazuhiro Hirase, Satoshi Shirahama

**Affiliations:** 1 Narao Medical Center, Shinkamigoto, Japan; 2 Kamigoto Hospital, Shinkamigoto, Japan; 3 Department of Digestive Disease Information & Research, Kurume University School of Medicine, Kurume, Fukuoka, Japan; 4 Division of Gastroenterology, Department of Medicine, Kurume University School of Medicine, Kurume, Fukuoka, Japan; 5 Arikawa Medical Center, Shinkamigoto, Japan; 6 Narao Medical Center, 712-3 Narao-go Shinkamigoto-cho Minamimatsuura-gun, Nagasaki, Japan

**Keywords:** Hepatitis C virus, Hepatocellular carcinoma, Prospective cohort study, Interferon, Life expectancy

## Abstract

**Background:**

No study has compared the long-term prognoses of hepatitis C patients with hepatitis C virus (HCV) antibody-negative individuals and investigated the effects of interferon (IFN) treatment. To clarify the long-term prognosis of HCV-positive residents of an isolated Japanese island and prospectively investigate the effects of IFN treatment in comparison with the HCV-negative general population.

**Methods:**

HCV antibody was positive in 1,343 (7.6%) of the 17,712 individuals screened. 792 HCV RNA-positive, HBsAg-negative subjects were enrolled. 1,584 HCV antibody-negative, HBsAg-negative general residents were sex- and age-matched to the 792 subjects. A total of 154 <70-year-old patients without liver cirrhosis (LC) or hepatocellular carcinoma (HCC) underwent IFN treatment. The survival rate with all-cause death as the endpoint was determined and causes of death were compared.

**Results:**

The 10- and 20-year survival rates of the hepatitis C and general resident groups were 65.4% and 87.8%, and 40.8% and 62.5%, respectively (*p* < 0.001; hazard risk ratio, 0.444; 95% confidence interval (CI): 0.389–0.507). There were 167 liver disease-related deaths and 223 deaths from other causes in the hepatitis C group, and 7 and 451, respectively, in the general resident group. Liver disease-related death accounted for 43.8% and 1.5% of deaths in the hepatitis C and general resident groups (*p* < 0.0001). The cumulative survival rate of the hepatitis C patients without IFN (n = 328) was significantly lower than the gender- and age-matched general resident group (n = 656) (*p* < 0.0001) but there was no significant difference between the IFN-treated (n = 154) and general resident groups (n = 308).

**Conclusions:**

In the hepatitis C group, the proportion of liver disease-related death was markedly higher, and the survival rate lower, than the general resident group. Introduction of IFN treatment in <70-year-old patients with hepatitis C without LC or HCC improved the survival rate to a level comparable to that of the general residents.

## Background

Chronic hepatitis C virus (HCV) infection rarely resolves spontaneously [1] and often causes chronic hepatitis [2], which is likely to lead to liver cirrhosis (LC) and hepatocellular carcinoma (HCC) [3-6]. HCC is a major cause of death associated with hepatitis C in Japan [7,8]. Treatment with interferon (IFN), introduced clinically in the early 1990s, normalizes transaminase levels [9,10], eradicates HCV [11,12] and improves liver fibrosis [13-15]. Eradication of the virus has been reported to reduce HCC occurrence [6,16]. Overall, IFN treatment clearly reduces the rate of liver disease-related death and improves life expectancy [17-19]. However, no study has investigated prospectively the long-term prognosis and final outcomes of hepatitis C by comparing life prognosis with death from all causes as the endpoint using the HCV antibody-negative general population as a control group.

In this study, we examined the life prognosis and the extent of improvement of life expectancy in hepatitis C patients with IFN treatment, using the general residents of an isolated island in Nagasaki Prefecture, Japan, as a control group. Because the subjects were residents of this island, immigration and emigration were relatively rare; therefore, it was easier to evaluate final outcomes and survival status. From this perspective, the number of individuals with unspecified final outcomes was expected to be minimized, and thus, the long-term clinical courses of hepatitis C patients would be elucidated. Furthermore, how IFN treatment improved the life prognosis of hepatitis C patients toward that of the general population of the island was investigated.

## Patients and methods

### Patients

**Figure 1:**
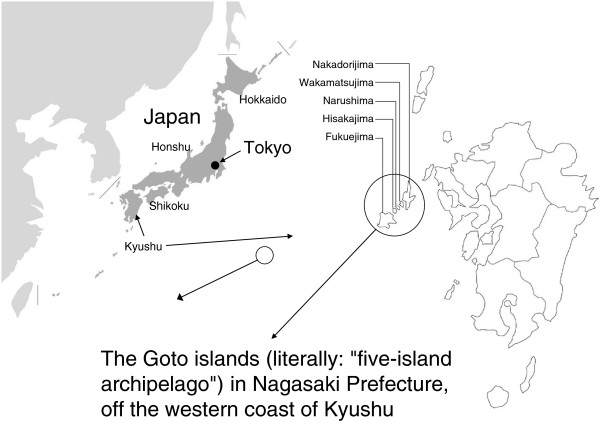
**Research area.** The subjects resided in a district located in the northern region of the Goto islands in Nagasaki prefecture in western Japan.

The subjects of this study were residents of Kamigoto island at the north part of the Goto archipelago, Nagasaki prefecture, which is located in western Japan (total population, 23,665 in 2007) (Figure 1). The Goto archipelago consists of about 140 islands, with the following five large islands (“Goto” literally means five islands) from the north to the south: Nakadori-jima, Wakamatsu-jima, Naru-shima, Hisaka-jima and Fukue-jima. These islands are located at the eastern part of the East China Sea. The southwestern archipelago with Fukue-jima as the center is called Shimogoto (southern part of the five islands), while the northeastern archipelago with Nakadori-jima as the center is termed Kamigoto (northern part of the five islands).

**Figure 2:**
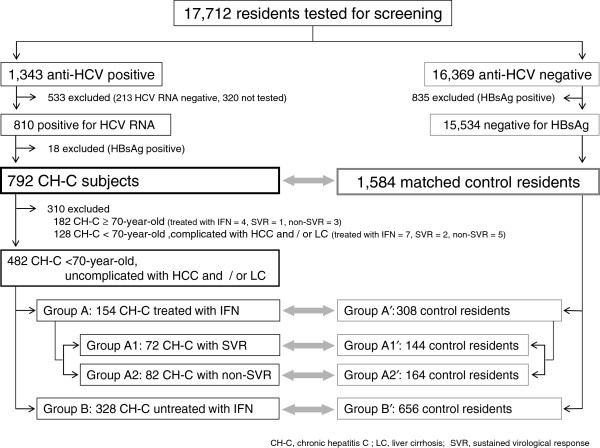
Schema for the subjects.

We initiated hospital-funded, free screening for HCV antibody prevalence (by ELISA) of subjects who visited the medical institute or underwent medical check-ups in January 1990. As of March 2007, 17,712 individuals had been tested. Of these, 1,343 (7.6%) were positive for HCV antibody and 1,023 (76.2%) underwent the secondary work-up test. After the exclusion of HBsAg-positive individuals, 792 patients positive for HCV RNA were enrolled in this study as the hepatitis C group (Figure 2). All people invited to take part in this prospective cohort study agreed to participate.

Meanwhile, from the 15,534 subjects remaining after the exclusion of HBsAg-positive individuals from the 16,369 negative for HCV antibody in the screening test, two local residents per hepatitis C group member were chosen for the general resident group (1,584 subjects) as sex- and age-matched controls. It should be noted that local residents who had their birthdays close to those of the patients in the hepatitis C group were chosen sequentially from the general resident group so that ages at the time of the follow-up would closely match between the two groups.

### Interferon therapy

IFN treatment was introduced in April 1992, when it was approved by the national insurance in Japan. We encouraged patients who were <70 years of age and had persistent abnormal transaminase levels (>40 IU/mL)**,** but without LC or HCC**,** to receive IFN treatment. Treatment was performed only when informed consent was obtained.

Liver biopsy was carried out within four weeks of the start of IFN treatment for histological evaluation. Liver biopsy specimens were assessed according to the criteria of Desmet et al., [20] where the staging of fibrosis was defined as F0 (no fibrosis), F1 (mild fibrosis), F2 (moderate fibrosis), F3 (sever fibrosis), or F4 (cirrhosis). Natural IFN-α, recombinant IFN-α 2a, IFN-α2b or IFN-β was selected for IFN monotherapy and given for 8 to 24 weeks. Since 2002, a combination of IFN and ribavirin has been given for 24 to 48 weeks.

IFN was used in 165 cases up to November 2010: 161 were younger than 70 years, and 4 were 70 years or older. Of these 165 cases, 135 and 30 underwent IFN treatment for the first time and as retreatment, respectively. HCV RNA was persistently negative for six months or longer after the end of the treatment (i.e., sustained virological response, SVR) in 75 cases (45.5%).

Of the 792 patients in the hepatitis C group, 182 were 70 years or older, and 610 were younger than 70 years. As shown in Figure 2, the 482 cases younger than 70 years without LC or HCC were categorized into subgroups and the efficacy of IFN treatment was evaluated separately. The subgroup composition was as follows: Group A, 154 patients who underwent IFN treatment; Group A1, 72 with SVR; Group A2, 82 with non-SVR; and Group B, 328 without IFN treatment.

Two local residents who were sex- and age-matched to each member of subgroups A, A1, A2, and B in the hepatitis C group were chosen. The subgroups of the general residents were designated as A′ (n = 308), A1′ (n = 144), A2′ (n = 164), and B′ (n = 656), respectively.

### Follow-up and diagnosis of HCC

The day of the screening test for HCV antibody was designated the starting day for follow-up. Subjects were followed-up until August 2011. In addition, in the analysis of the efficacy of IFN therapy, the day of introduction of IFN therapy was the starting day of follow-up of groups A, A1, A2, and B. LC was diagnosed by liver biopsy and laparoscopy. LC was diagnosed when typical images for LC were observed by abdominal ultrasonography and/or CT, and esophageal or gastric varices were concomitantly observed endoscopically.

Imaging diagnosis of HCC was made at least twice per year. Space-occupying lesions detected or suspected at the time of ultrasonography were further examined with computed tomography, Magnet resonance imaging system, selective hepatic angiography, and fine-needle aspiration biopsy. Clinical trends of tumor markers were also taken into account. A final diagnosis of HCC was based on histological findings from resected hepatic tumors or biopsy specimens, or on the radiological findings of selective hepatic angiography.

## Ethics considerations

The test results were explained to the subjects after informed consent was obtained. The study protocol was approved by the Ethics Committee of Kamigoto Hospital (reference number: 06–03) in accordance with the Declaration of Helsinki.

### Statistical analysis

Statistical analysis was performed using SPSS version 19.0J for Windows. The level of statistical significance was set at *p* < 0.05. Continuous variables are expressed as mean ± standard deviation. Differences of means were evaluated using Student’s *t*–test or the Mann–Whitney *U*–test. Discrete variables were analyzed using the chi-squared test. Cumulative survival curves were determined using the Kaplan–Meier method. The values of the confidence intervals (CIs) indicate the lower and upper 95% confidence limits.

## Results

### Baseline patient characteristics

Table 1 shows the backgrounds of the 792 cases in the hepatitis C group and 1,584 cases of the general resident group. At the first visit, LC was observed in 177 (22.3%) and 8 cases (0.4%) in the hepatitis C and general resident groups, respectively; HCC was present at the first visit in 45 (5.7%) and 2 cases (0.1%), respectively. Both diseases were diagnosed significantly more often in the hepatitis C group (*p* < 0.001). IFN treatment was introduced during the follow-up in 165 cases (20.8%) in the hepatitis C group and in no cases in the general resident group.

**Table 1:** Demographic characteristics of the patients

	**Hepatitis C (n = 792)**	**Control (n = 1584)**	**Difference**
Male: Female	512: 280	1024: 560	matched
Birth year −1929	320	640	matched
−1939	279	558	matched
1940-	193	386	matched
Age at first visit (mean ± SD), years	60.9 ± 12.3	60.9 ± 12.3	matched
LC at first visit	177 (22.3%)	8 (0.4%)	< 0.001
HCC at first visit	45 (5.7%)	2 (0.1%)	< 0.001
Liver biopsy/laparoscopy (F0–1 : F2 : F3 : F4)	446 (56.3%) (182 : 83 : 42 : 139)		
IFN administration	165 (20.8%)	0 (0%)	
SVR	75 (45.5%)		
Non-SVR	90 (54.5%)		
Outcomes Survival	327 (41.3%)	1031 (65.1%)	< 0.0001
Death	400 (50.5%)	482 (30.4%)	
Unknown	65 (8.2%)	71 (4.5%)	

During the follow-up, laparoscopy, liver biopsy, or both were carried out in 446 cases (56.3%) in the hepatitis C group. HCV serogroups or genotypes were determined in 742 of the 792 cases (93.7%) in the hepatitis C group. Serotype 1 or genotype 1b was observed in 437 cases (58.9%), serotype 2 or genotypes 2a or 2b was recognized in 270 cases (36.4%), and serotypes or genotypes were unidentified in 35 cases (4.7%).

Survival, death, and unknown status at the final follow-up were observed in 327 (41.3%), 400 (50.5%), and 65 cases (8.2%) in the hepatitis C group and 1,031 (65.1%), 482 (30.4%), and 71 cases (4.5%) in the general resident group, respectively. The median follow-up periods were 11.5 years (maximum, 21.7 years) and 16.1 years (maximum, 21.7 years) for the hepatitis C and general resident groups, respectively.

### Survival rate

**Figure 3:**
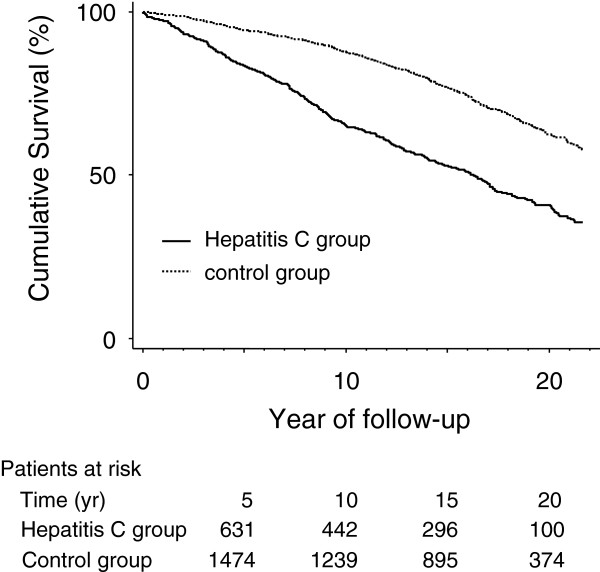
**Cumulative survival rates in the hepatitis C and control groups.** All-cause mortality was adopted as the endpoint and the results were analyzed using the Kaplan-Meier method. The hazard ratio was 0.444 (95% CI: 0.389–0.507). The solid and dotted lines indicate the hepatitis C and control groups, respectively.

With death from all causes as the endpoint, the survival rates in the hepatitis C and general resident group were 83.7% and 94.5% at 5 years, 65.4% and 87.8% at 10 years, 52.9% and 76.8% at 15 years, and 40.8% and 62.5% at 20 years, respectively. The survival rate was significantly lower in the hepatitis C group (*p* < 0.001, Figure 3). The hazard risk ratio was 0.444 (95% CI: 0.389–0.507).

### Causes of death

The causes of death of the 400 and 482 patients in the hepatitis C and general resident groups who died during the follow-up period were analyzed. In particular, the proportions of liver disease-related deaths such as death from HCC, liver failure, or gastrointestinal bleeding and other causes of death were compared between groups.

**Figure 4:**
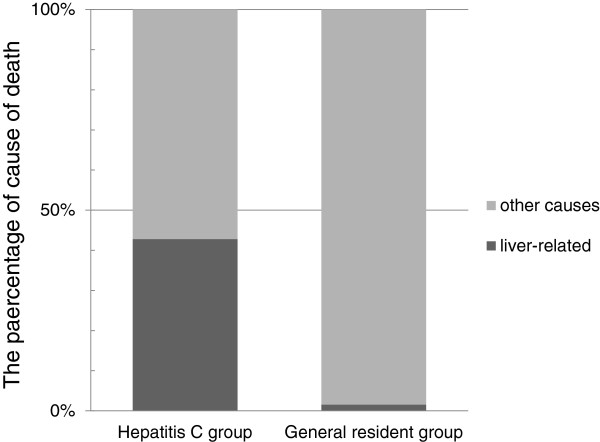
**Causes of death in the hepatitis C and general resident groups after excluding cases with indeterminate causes of death.** Liver disease-related deaths, such as deaths due to HCC, hepatic failure and gastrointestinal bleeding accounted for 42.8% (n = 167) of cases in the hepatitis C group and 1.5% (n = 7) of cases in the general resident group.

As shown in Figure 4, out of 390 cases excluding 10 (2.5%) with an unidentified cause of death out of 400 fatal cases in the hepatitis C group, there were 167 liver disease-related deaths (42.8%; 145 HCC, 19 liver failure, and 3 gastrointestinal bleeding) and 223 deaths from other causes (57.1%; 100 other malignant tumor, 36 pneumonia, 19 heart disease, 15 cerebral vascular disease, 53 others). On the other hand, out of 458 cases**,** excluding 24 (5.0%) with an unidentified cause of death from the 482 fatal cases in the general resident group, there were 7 liver disease-related deaths (1.5%; 5 HCC, 2 liver failure and 0 gastrointestinal bleeding) and 451 deaths from other causes (98.5%).

The average ages at the time of death were 73.2 ± 8.3 years (n = 279) in males and 78.2 ± 9.8 years (n = 121) in females in the hepatitis C group; those in the general resident group were 79.1 ± 8.6 years (n = 338) and 83.4 ± 8.8 years (n = 144), respectively. The average ages at the time of death were significantly lower for both sexes in the hepatitis group (*p* < 0.001).

### Interventional effect of IFN therapy

The A, A1, A2, and B subgroups, younger than 70 years and without LC or HCC, consisted of 482 cases in the hepatitis C group (n = 792), in which IFN treatment was actively introduced. The survival rates of these groups were compared to their respective age- and sex-matched subgroups (A′, A1′, A2′, and B′).

The background data of Groups A, A1, A2, and B are shown in Table 2. The proportions of males were significantly higher in Groups A, A1, and A2, in which IFN was introduced, than that in Group B, in which IFN was not introduced (*p* = 0.003, *p* = 0.013, and *p* = 0.045, respectively). There were no significant differences in age between the groups. ALT levels 40 IU/mL (the upper limit of normal) or lower were observed 95% of the time or more during the follow-up period in 2 (1.3%), 0 (0%), 2 (2.4%), and 129 (39.4%) cases in Groups A, A1, A2, and B, respectively. Such results were observed significantly more often in Group B than in all other groups (*p* < 0.001 in all comparisons).

**Table 2:** Patient backgrounds with and without IFN treatment

	**IFN treated Group A (n = 154)**	**SVR Group A1 (n = 72)**	**Non-SVR Group A2 (n = 82)**	**No treated Group B (n = 328)**
Male	117 (76.0%)	56 (77.8%)	62 (75.6%)	205 (62.5%)
Age at first visit (mean ± SD), years	56.9 ± 11.5	55.7 ± 12.6	58.0 ± 10.3	57.5 ± 10.4
ALT < 40 IU/mL	2 (1.3%)	0 (0%)	2 (2.4%)	129 (39.4%)

**Figure 5:**
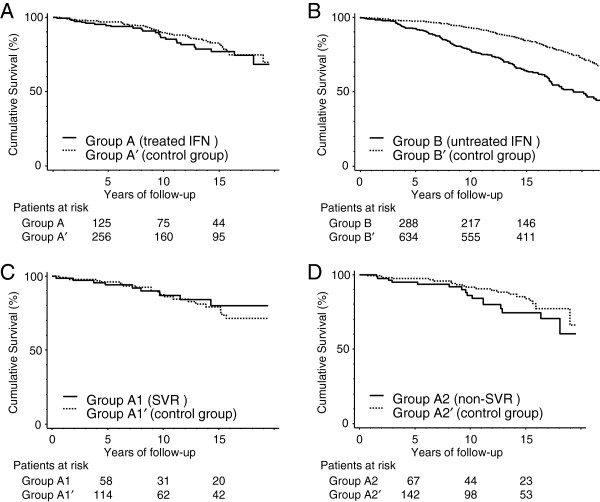
**Cumulative survival rates between corresponding groups.** (**A**) There was no significant difference between the IFN-treated hepatitis C group (Group A) and the general resident group (Group A^′^). (**B**) There was a significant difference between the hepatitis C group not receiving IFN treatment (Group B) and the general resident group (Group B^′^) (*p* < 0.0001). (**C**) There was no significant difference between the hepatitis C group that achieved SVR by IFN treatment (Group A1) and the general resident group (Group A1^′^). (**D**) There was no significant difference between the IFN treatment group with non-SVR in the hepatitis C group (Group A2) and the general resident group (Group A2^′^) (*p* = 0.153). All survival rates were calculated using the Kaplan–Meier method. Solid and dotted lines represent the hepatitis C and general resident groups, respectively.

The cumulative survival rates in Groups A, A1, A2, and B in the hepatitis C group were compared to their corresponding groups in the general resident group (Figure 4). In Groups A and A′, the 5-, 10-, and 15-year survival rates were 94.5% and 96.9%, 86.5% and 89.8%, and 76.9% and 82.0%, respectively. Irrespective of the effect of treatment, there was no significant difference in the survival rates between cases receiving IFN therapy and the general resident group (Figure 5A).

In Groups A1 and A1′, the 5-, 10-, and 15-year survival rates were 94.0% and 96.3%, 87.1% and 87.4%, and 80.1% and 79.2%, respectively. The survival rates were not significantly different between groups (Figure 5C). In Groups A2 and A2′, the 5-, 10-, and 15-year survival rates were 95.1% and 97.5%, 86.1% and 91.6%, and 74.6% and 83.8%, respectively. The survival rates were lower in Group A2, but the difference was not significant (*p* = 0.153, Figure 5D).

In Groups B and B′, the 5-, 10-, and 15-year survival rates were 92.4% and 97.4%, 77.7% and 93.0%, and 64.2% and 84.6%, respectively. The survival rates were significantly lower in Group B (*p* < 0.001, Figure 5B).

The causes of death were compared specifically in cases with ALT levels of 40 IU/mL or more during the follow-up period in Groups A, A1, A2, and B, and the influence of IFN therapy on the cause of death was examined (Table 3). During the follow-up period, there were 25, 9, 16, and 90 deaths in Groups A, A1, A2, and B, respectively. Among the deaths, liver disease-related deaths were observed in 6 (24%), 1 (11.1%), 5 (31.2%), and 32 cases (36.0%) in Groups A, A1, A2, and B, respectively, excluding cases with unknown causes of death (0, 0, 0, and 1 cases, respectively). Despite the lack of significant differences, the proportion of liver disease-related death was lower in Group A1 than in Group B.

**Table 3:** Causes of death in the hepatitis C group with abnormal transaminase levels

	**IFN treated Group A (n = 152)**	**SVR Group A1 (n = 72)**	**Non-SVR Group A2 (n = 80)**	**No treated Group B (n = 199)**
Total deaths	25	9	16	90
Unknown cause	0	0	0	1
Liver-related	6 (24.0%)	1 (11.1%)	5 (31.2%)	32 (36.0%)
Others	19 (76.0%)	8 (88.9%)	11 (68.8%)	57 (64.0%)

## Discussion

In this study, we compared prospectively the life expectancy and final outcomes of all hepatitis C patients detected by screening with sex- and age-matched HCV antibody-negative general residents in a specific area. The median follow-up period exceeded 10 years, the follow-up survey was excellent in that only 10% of cases had unknown final outcomes.

As mentioned above, because the subjects were residents of an isolated island, where immigration and emigration were relatively rare, the follow-up was quite successful. Moreover, environmental factors, such as medical care, were comparable between the two groups as a result of approximating birth date in the same residential district when choosing general residents who matched the subjects in the hepatitis C group. Furthermore, the accuracy of the comparative study in cases with and without persistent HCV infection was considered to be excellent.

Hepatocarcinogenesis can be prevented in hepatitis C when HCV is persistently undetectable and transaminases are normalized by IFN treatment [6,21-23]. In addition, it is reported that IFN treatment improves the life prognosis in hepatitis C [17,18,24-27]. However, no report has prospectively examined the effect of IFN treatment with HCV antibody-negative general residents as controls; to our knowledge, the present study is the first of its kind.

In terms of the overall prognosis of hepatitis C, the survival rate was lower than in the HCV antibody-negative general residents; the hazard ratio was 0.444 (95% CI: 0.389–0.507). Liver disease-related death accounted for 42.8% of all causes of death in the hepatitis C group; this greatly exceeded the 1.5% for the general residents. The survival rate in the patients persistently infected with HCV was markedly low and their prognosis was extremely poor.

We then determined to what extent IFN treatment improved the prognosis of the hepatitis C group. The patients younger than 70 years who had indications for IFN treatment and had no LC or HCC were compared to the general resident group. When SVR was obtained by IFN treatment, the prognosis in the hepatitis C group was comparable to that in the general resident group. Kaplan–Meier analysis showed almost overlapping curves for the two groups. In cases with non-SVR, despite the slightly lower survival rates in the general resident group, the prognosis in the hepatitis C group was not significantly different. Taken together, irrespective of the effect of IFN treatment, the survival rate of members of the hepatitis C group who received IFN treatment was comparable to that of the general resident group.

Yoshida et al. [18] reported that the standardized mortality rate from all causes was 1.4 (0.8–2.3) in a non- LC group without IFN treatment compared to 0.3 (0.1–0.7) in an SVR group after IFN treatment and 0.7 (0.5–1.1) in a non-SVR group after IFN treatment; these results are consistent with ours. It may be reasonable to conclude that to a certain extent, intervention with IFN treatment eliminates the risk of hepatocarcinogenesis due to persistent long-term infection with HCV. Furthermore, achieving SVR eliminates the risk almost completely. Even in cases with non-SVR, the risk of hepatocarcinogenesis was reduced and the prognosis became closer to that of the general resident group.

Regarding the results obtained using death from all causes as the endpoint, we presumed the following: First, the group receiving IFN treatment had a higher awareness of health management and continued periodical medical check-ups and clinic visits with a greater frequency than the group without IFN treatment. Second, we presumed that this higher awareness of health management had a preventive effect against a variety of diseases in addition to liver disease and enabled effective treatment following early disease detection.

In this study, IFN treatment introduction was not assigned randomly; therefore, there was a difference in the backgrounds between the two groups. There was a bias in that the IFN–treated group had a higher proportion of males and more cases with ALT values exceeding the normal range. In general, male sex and high transaminase levels are risk factors for hepatocarcinogenesis; the survival rate in such cases is low [2,7,8]. However, even in such cases with a high risk for hepatocarcinogenesis, introduction of IFN treatment reduced the mortality from HCC and improved the survival rate; furthermore, the prognosis was comparable to that in the general resident group.

Yoshida et al. reported that IFN treatment improved the prognosis of hepatitis C in a retrospective cohort study [18]. Although their conclusion is consistent with ours, our study is a prospective cohort study, and the median of the follow-up period exceeded 10 years. Moreover, our study is unique in that it is a comparative study with HCV antibody-negative general residents of the same district in the same time period as controls.

Nagao et al. carried out a survey of patients infected with HCV in a district of Japan and reported that the factors that influence the acceptance of IFN treatment are the clinics the patients visited, sex, and the presence or absence of concomitant disease [28]. Because patients infected with HCV have been getting older in Japan, cooperation between liver specialists and non-experts in individual districts is important to introduce IFN treatment as early as possible, particularly for people under 70 years.

## Conclusion

We compared the final outcomes and prognosis between all patients with hepatitis C and HCV antibody-negative general residents in the district. In conclusion, persistent infection with HCV led to a markedly poor prognosis, but introduction of IFN treatment before the age of 70 years reduced the risk associated with HCV infection and improved the prognosis to a level comparable to that of the general population.

## Abbreviations

HCV: Hepatitis C virus; CH-C: Chronic hepatitis C; LC: Liver cirrhosis; HCC: Hepatocellular carcinoma; IFN: Interferon; PCR: Polymerase chain reaction; SVR: Sustained virological response; ALT: Alanine aminotransferase.

## Competing interests

The authors declare that they have no competing interests.

## Authors’ contributions

Study concept and design, analysis and interpretation of data, and drafting of the manuscript: KY; acquisition of data: MT, TS, and KH; drafting of the manuscript: YN; revised the article critically for important intellectual content: MS, SS. All authors have read and approved the submitted manuscript.

## Financial support

This study was supported in part by Health and Labour Sciences Research Grants for Research on Hepatitis from the Ministry of Health, Labour and Welfare of Japan (H20-kannen-004).

## Pre-publication history

The pre-publication history for this paper can be accessed here:

http://www.biomedcentral.com/1471-230X/12/139/prepub
